# Dual RNA-Seq Unveils Candidate Key Virulence Genes of *Vibrio harveyi* at the Early Stage of Infection in Hybrid Grouper (♀ *Epinephelus polyphekadion* × ♂ *E. fuscoguttatus*)

**DOI:** 10.3390/microorganisms12112113

**Published:** 2024-10-22

**Authors:** Yan-Hua Zeng, Wen Li, He Xu, Xiao-Xiao Gong, Yu-Mei Zhang, Hao Long, Zhen-Yu Xie

**Affiliations:** 1 School of Marine Biology and Fisheries, Hainan University, Haikou 570228, China; zengyanhua@hainanu.edu.cn (Y.-H.Z.); liw2112@163.com (W.L.); 18669856227@163.com (H.X.); 18208986182@163.com (X.-X.G.); 18783907506@163.com (Y.-M.Z.); longhao@hainanu.edu.cn (H.L.); 2 State Key Laboratory of Marine Resource Utilization in South China Sea, Hainan University, Haikou 570228, China; 3 Hainan Provincial Key Laboratory for Tropical Hydrobiology and Biotechnology, Hainan University, Haikou 570228, China

**Keywords:** *Vibrio harveyi*, dual RNA-seq, hybrid grouper, virulence factor, host–pathogen interactions

## Abstract

*Vibrio harveyi* is a major bacterial pathogen that causes disease in aquaculture animals worldwide. Although *V. harveyi* consistently harbors a range of traditional virulence genes, it remains unclear which specific genes are crucial for virulence at different infection stages. Dual RNA-seq is a cutting-edge RNA sequencing technology that is ideal for investigating the gene expression patterns of pathogens within the host, which is highly effective in identifying key virulence genes. In previous artificial infection experiments, we have identified the liver of hybrid grouper (♀ *Epinephelus polyphekadion* × ♂ *E. fuscoguttatus*) as the main target organ for pathogenic *V. harveyi* GDH11385 during the initial infection phase. To further explore the key virulence factors of *V. harveyi* at the early stage of infection, the liver of the hybrid grouper infected with strain GDH11385 was analyzed here by dual RNA-seq. The transcriptome data were compared with that of in vitro cultured bacteria. The results showed that 326 and 1140 DEGs (differentially expressed genes) were significantly up- and down-regulated, respectively, at 4 h post-infection (hpi). Further pathway enrichment analyses revealed that these up-regulated DEGs in vivo were mainly enriched in siderophore biosynthesis and transport, type VI secretion system (T6SS), flagellar assembly, glycolysis/gluconeogenesis, and ribosome. Notably, all genes involved in the metabolism and utilization of vibrioferrin (a carboxylate class of siderophore produced by *Vibrio*), and most of the genes within one of three T6SSs, were significantly up-regulated in vivo. This indicates that siderophore-dependent iron competition and T6SS-mediated delivery of virulence factors are vital for the successful colonization of *V. harveyi* at the early stage of infection. This study provides more precise clues to reveal the virulence mechanism of *V. harveyi* during the initial phase of infection.

## 1. Introduction

*Vibrio harveyi* is the most common bacterial pathogen affecting tropical and subtropical mariculture animals, causing serious damage to the global mariculture industry and, in particular, to the aquaculture industry in South China [1,2]. It has been shown that *V. harveyi* is responsible for causing vibriosis in nearly 70% of groupers (*Epinephelus* sp.), an economically valuable fisheries species in South China [3,4]. *V. harveyi* has also been reported to seriously affect the shrimp farming industry in countries such as China, India, Malaysia, Thailand, and the Philippines [5,6]. We have recently found that the median lethal dose (LD_50_) of pathogenetic *V. harveyi* isolated from diseased fish in Hainan Province was significantly lower than previously reported. Infected fish displayed prominent symptoms such as open mouth, scale loss, fin ulceration, and body ulceration (muscle rot), notably with severe caudal fin ulceration being the most prevalent [1,7,8]. These observations suggest that the virulent strains of *V. harveyi* currently circulating in Hainan Province exhibit higher levels of virulence compared to those found in other regions.

The pathogenicity of *Vibrio* strains relies on a wide range of virulence genes, such as those involved in virulence factor production, nutrient acquisition, cell motility and adhesion, environmental response, and quorum sensing [9,10]. For example, Zhang et al. correlated the high virulence of *V. harveyi* VIB 645 to salmonids with the presence of duplicate hemolysin genes (*vhhA* and *vhhB*), as the majority of the less virulent or avirulent isolates had single genes or none at all [11]. Soto-Rodriguez et al. reported that the virulence of luminous *V. harveyi* to *Artemia franciscana* nauplii was highly correlated with the production of proteases, phospholipases, or siderophores [12]. Furthermore, the virulence of *V. harveyi* ORM4 towards the European abalone *Haliotis tuberculata* has been shown to involve both quorum sensing and a type III secretion system [13]. However, most studies on the virulence mechanisms of *V. harveyi* have focused only on specific genes, and thus it remains challenging to identify the critical virulence genes essential for infecting the host.

Bacterial virulence is largely dependent on the strength of the host immune defense. The virulent strains may not cause disease in hosts with strong immunity, whereas avirulent isolates can be pathogenic for immunocompromised hosts [14,15]. When *V. harveyi* infects the host, it is highly likely to exhibit a unique virulence gene expression pattern that remains undiscovered. Therefore, studying the variation in gene expression patterns of *V. harveyi* in the host compared to in vitro culture is the most direct and efficient approach to comprehending its pathogenic mechanisms. Dual RNA-seq is an innovative RNA-sequencing method that simultaneously detects the transcriptional profiles of both pathogen and host during infection, providing insights into the pathogenic mechanisms and host immune responses [16]. Recently, several key virulence genes have been identified in the virulent *Pseudomonas plecoglossicida* during infection of orange-spotted grouper (*Epinephelus coioides*) or large yellow croaker (*Larimichthys crocea*) using dual RNA-seq. These genes include *dksA* (encoding an RNA polymerase-binding transcription factor), *secY* (encoding an integral membrane protein), the flagellar gene *fliN*, a transcriptional regulator gene, and an ABC transporter gene [17,18,19,20]. The application of dual RNA-seq to identify key virulence genes in *V. harveyi* during host infection is not currently reported.

The hybrid grouper (♀ *Epinephelus polyphekadion* × ♂ *E. fuscoguttatus*) represents a newly developed species of artificial grouper extensively cultivated in South China [21]. Hainan Province, a key site for this aquaculture, boasts an annual production of 100,000 tons. Despite this significant output, the survival rate of hybrid groupers in Hainan is highly variable, primarily due to outbreaks of bacterial diseases, with *V. harveyi* being particularly prevalent. In previous artificial infection experiments, we have identified the liver of hybrid grouper as the main target organ for pathogenic *V. harveyi* GDH11385 during the initial infection phase [8]. Herein, the livers of hybrid groupers infected with pathogenic *V. harveyi* GDH11385 were subjected to dual RNA-seq. By comparing the differences in gene expression patterns of *V. harveyi* in the host versus in vitro culture (grown in culture medium), the crucial virulence genes of *V. harveyi* during the early stage of infection were identified. Our study shows that dual RNA-seq is a rapid and potent tool for identifying crucial virulence factors and understanding intricate pathogenic processes during various stages of infection.

## 2. Materials and Methods

### 2.1. Bacterial Strains and Culture Conditions

The pathogenic *V. harveyi* strain GDH11385 was isolated from diseased *Epinephelus coioides* in a mariculture system and was shown to be highly virulent to healthy hybrid groupers by an artificial infection assay [1]. Strain GDH11385 was routinely cultured in LBS liquid medium at 30 °C with oscillation at 180 rpm. The LBS medium consisted of 30 g/L NaCl, 5 g/L yeast extract, and 10 g/L peptone.

### 2.2. Artificial Infection and Sampling

The healthy hybrid groupers (♀ *Epinephelus polyphekadion* × ♂ *E. fuscoguttatus*) with a body weight of 12 ± 1.0 g were purchased from Hainan Lantaibang Biotechnology Co., Ltd. (Wenchang, China). Prior to infection, the fish were accommodated in filtered and disinfected seawater for two weeks under experimental conditions at 29 ± 1 °C, with the salinity of the water maintained at 30‰. The acclimatized healthy fish were divided into two groups, each consisting of three 200-L tanks, with each tank holding 20 fish and 140 L of filtrated seawater. For the experimental group, each fish was intraperitoneally injected with 200 µL of bacterial suspension of strain GDH11385 at a concentration of 1 × 10^7^ CFU/mL [1]. For the negative control group, each fish was injected with 200 µL of sterile PBS buffer. For the tissue dual RNA-seq assay, liver samples were collected from three fish infected with strain GDH11385 or treated with PBS buffer at 4 h post-infection (hpi). This time point was chosen based on prior observations that the concentration of GDH11385 in the liver peaked at 4 hpi [8]. For the in vitro cultivation group, strain GDH11385 was grown in LBS liquid medium at 30 °C with shaking at 180 rpm for 24 h. Three biological replicates per group were prepared, immediately frozen in liquid nitrogen, and then stored at −80 °C until RNA extraction.

### 2.3. RNA Extraction, Library Construction, and Sequencing

Total RNA was extracted from the lives of infected hybrid groupers and in vitro cultured *V. harveyi* GDH11385 using the TRIzol reagent (Vazyme, Nanjing, China) following the manufacturer’s instructions. The NanoDrop 2000 spectrometer (Thermo Fisher Scientific, Waltham, MA, USA) was used to determine RNA concentration and purity, while RNA integrity was evaluated with agarose gel electrophoresis and an Agilent 2100 Bioanalyzer (Agilent Technologies, Santa Clara, CA, USA). The rRNA in total RNA was removed using the Ribo-Zero rRNA Removal Kit (Epicentre, Madison, WI, USA).

RNA-seq libraries were prepared following a standardized protocol using an Illumina TruSeq RNA Sample Prep Kit (San Diego, CA, USA). In brief, rRNA-depleted RNA samples underwent fragmentation in a fragmentation buffer, followed by cDNA synthesis using a SuperScript Double-Stranded cDNA Synthesis Kit (Invitrogen, Carlsbad, CA, USA). Subsequently, end reparation, phosphorylation, and poly (A) addition were performed to prepare the cDNA libraries, which were then amplified using Phusion DNA polymerase. To ensure library quality, an Agilent 2100 Bioanalyzer was employed for assessment. Finally, the libraries were subjected to high-throughput sequencing on an Illumina NovaSeq X Plus sequencer (150 bp × 2 read length) at Majorbio Biotech Co., Ltd. (Shanghai, China).

### 2.4. Sequencing Data Processing and Reads Mapping

The raw sequencing reads were subjected to quality control by removing adapter contamination, trimming low-quality reads, and correcting wrongly represented bases by fastp (version 0.19.7) [22]. The high-quality sequences obtained were then compared to ribosomal RNA (rRNA) sequences in the Rfam database (https://rfam.org/ (accessed on 21 October 2023)), and rRNA reads were removed prior to further analyses. Clean data were mapped to the reference genome of *V. harveyi* GDH11385 using Bowtie2 [23]. The data were analyzed via the online Majorbio Cloud Platform (https://cloud.majorbio.com (accessed on 1 November 2023)) [24].

### 2.5. Identification of Differentially Expressed Genes (DEGs)

The expression level of each mRNA transcript is normalized by transcripts per million (TPM) using the RSEM software (version 1.3.3) [25]. Venn analysis was performed to determine the genes that were commonly and uniquely expressed in *V. harveyi* GDH11385 under in vivo and in vitro conditions. Based on the expression level of all genes, principal component analysis (PCA) was performed using an R package (ggplot2 v3.4.0). The DESeq2 software (v3.10) was used to screen for differentially expressed genes (DEGs) based on the criteria of *p*-adjust < 0.05 and |log_2_FC| ≥ 1 [26]. The Benjamini-Hochberg procedure was used to adjust p-values when performing multiple testing to control for false discovery rates [27]. The DEGs were annotated for function using the NR (https://www.ncbi.nlm.nih.gov/ (accessed on 15 November 2023)), Swiss-Prot (http://www.expasy.ch/sprot/ (accessed on 20 November 2023)), Pfam (http://pfam.xfam.org/ (accessed on 22 November 2023)), GO (https://www.geneontology.org/ (accessed on 24 November 2023)), and KEGG (https://www.kegg.jp/ (accessed on 26 November 2023)) databases.

### 2.6. Functional Enrichment Analysis

GO and KEGG pathway enrichment analysis was conducted to determine the key biological functions of DEGs in *V. harveyi* GDH11385 during infection of hybrid groupers. The Goatools (version 0.6.5) and KOBAS 2.0 web servers were utilized for GO and KEGG enrichment analysis, respectively [28,29]. A significant enrichment in the GO term or KEGG pathway was identified when the corrected p value (*p*-adjust) was less than 0.05.

## 3. Results

### 3.1. Sequencing Quality Assessment

An average of 53,159,245 clean reads were generated for each sample, with the Q20 and Q30 values of the sequencing data ranging from 97.42 to 98.52% and 94.09 to 95.51%, respectively (Table 1). The genome mapping ratio of the 4 hpi liver sample from infected groupers (group T4h) to the reference genome of *V. harveyi* GDH11385 was between 0.06% and 0.07%, which is within the acceptable range as previously documented [20,30]. For the in vitro cultured GDH11385, the genome mapping ratio ranged from 98.51% to 98.59% (Table 1). These results indicate that the sequencing quality is reliable and meets the requirements for subsequent data analysis.

### 3.2. Correlation Analysis of Gene Expression between Groups

**Figure 1:**
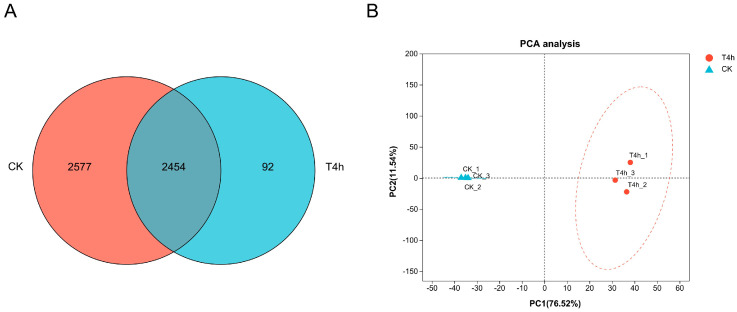
Correlation analysis of gene expression between two experimental groups. (**A**) Venn diagram illustrating the commonly and uniquely expressed genes in *V. harveyi* GDH11385 in the 4 hpi liver (T4h) and in the in vitro bacterial pure culture control (CK). (**B**) PCA analysis based on the expression level of all genes showing the correlation of gene expression between groups. Values in parentheses on the axes represent the percentage of the total variance explained by each principal component. Samples from the same group are represented by the same color and shape, where the red circle represents the 4 hpi liver sample (T4h) and the blue triangle corresponds to the in vitro bacterial pure culture control (CK).

Venn analysis showed significant differences in gene expression in *V. harveyi* during infection of groupers in comparison to in vitro culture. Of the 5905 genes in the genome of strain GDH11385, 2546 genes were commonly expressed in both the in vitro culture condition and the 4 hpi liver, while 2577 genes were exclusively expressed in the in vitro culture condition, and 92 genes were uniquely expressed in the 4 hpi liver (Figure 1A). It was noteworthy that only 42.79% (2546 genes) of the whole genome of strain GDH11385 was detected in the 4 hpi liver. PCA analysis showed consistency within duplicate samples of the same experimental group and significant differences across different groups. The first (PC1) and second (PC2) principal components explained 76.52% and 11.54% of the variation, respectively (Figure 1B). These results highlighted the distinct gene expression patterns of *V. harveyi* during infection as opposed to its growth in pure culture in vitro.

### 3.3. Analysis of Differentially Expressed Genes (DEGs)

**Figure 2:**
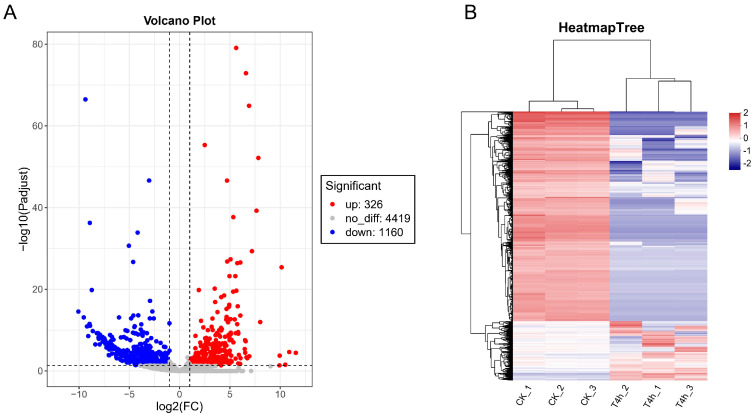
The DEGs visualized by volcano plot and hierarchical clustering heatmap. (**A**) Volcano plot showing the differences in expression profiles of strain GDH11385 between two experimental groups. Each dot in the graph represents a gene: red dots signify up-regulated genes, blue dots signify down-regulated genes, and black dots indicate genes that are not significantly different. (**B**) Hierarchical clustering heatmap tree of DEGs.

To understand how gene expression of *V. harveyi* changed during infection of hybrid groupers compared to in vitro cultivation, we used DESeq2 to screen for differentially expressed genes (DEGs) in strain GDH11385 as it propagated in vivo within the host compared to its growth in vitro. The volcano plot showed that 326 genes were significantly up-regulated and 1140 genes were significantly down-regulated at 4 hpi (Figure 2A). The DEGs were also visualized as a hierarchical clustering heatmap in which all samples clustered into their respective experimental and control groups, indicating a clear separation of gene expression between two groups (Figure 2B). The number of DEGs accounted for 25.2% of the whole genome of strain GDH11385, suggesting that the infection process triggers dynamic changes in the gene expression profiles in *V. harveyi*.

### 3.4. Functional Enrichment Analysis of DEGs

**Figure 3:**
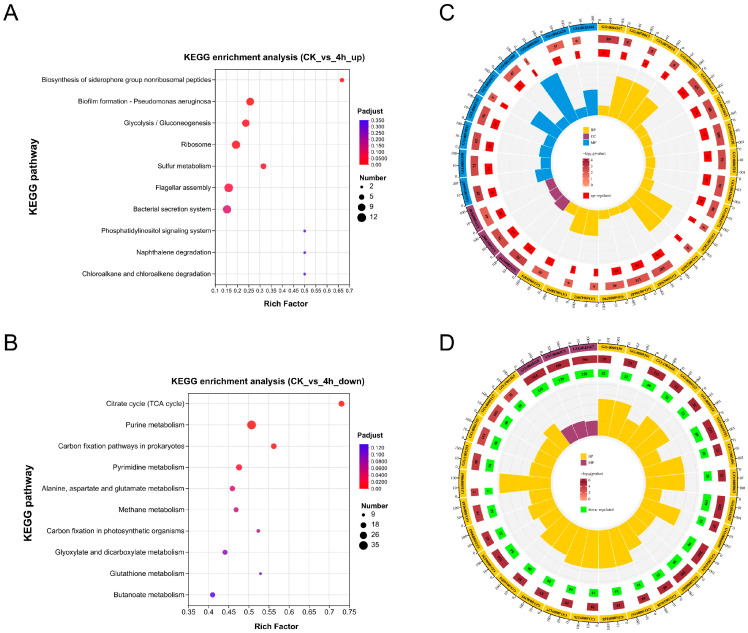
Functional enrichment analysis of DEGs. (**A**,**B**) show the KEGG pathway enrichment results for up-regulated and down-regulated DEGs, respectively. (**C**,**D**) display the GO enrichment circles of up-regulated and down-regulated DEGs, respectively.

The DEGs were subjected to functional enrichment analysis by mapping to the KEGG and GO databases to determine their biological functions. KEGG pathway enrichment analysis showed that the up-regulated DEGs in vivo were significantly enriched in seven pathways, including biosynthesis of siderophore group nonribosomal peptides, biofilm formation, glycolysis/gluconeogenesis, ribosome, sulfur metabolism, flagellar assembly, and bacterial secretion system (*p* < 0.01) (Figure 3A). The down-regulated DEGs in vivo were significantly enriched in ten pathways, most of which were related to metabolism, including citrate cycle (TCA cycle), purine metabolism, carbon fixation pathways in prokaryotes, pyrimidine metabolism, alanine, aspartate, and glutamate metabolism, methane metabolism, carbon fixation in photosynthetic organisms, glyoxylate and dicarboxylate metabolism, glutathione metabolism, and butanoate metabolism (*p* < 0.01) (Figure 3B).

Based on GO enrichment analysis of up-regulated DEGs, among the top 30 significantly enriched GO terms, 18 were affiliated with biological processes (BP), 9 with molecular functions (MF), and 3 with cellular components (CC). The most enriched GO term was cellular protein metabolic process (GO:0044267), followed by oxidoreductase activity, acting on a sulfur group of donors (GO:0016667) and cell outer membrane (GO:0009279) (Figure 3C). For the down-regulated DEGs, 27 of the top 30 significantly enriched GO terms belonged to the BP category, while the remaining 3 belonged to the MF category. Similar to the KEGG enrichment results, most of the enriched GO terms for down-regulated DEGs were associated with metabolism, e.g., the most enriched GO terms were ribonucleoside monophosphate biosynthetic process (GO:0009156), ribonucleoside monophosphate metabolic process (GO:0009161), alpha-amino acid metabolic process (GO:1901605), nucleoside monophosphate metabolic process (GO:0009123), and nucleoside monophosphate biosynthetic process (GO:0009124) (Figure 3D). These findings highlight a significant down-regulation in metabolic pathways in the analyzed dataset. These findings highlight the significant down-regulation of metabolic pathways of *V. harveyi* within the host.

### 3.5. Identification of Candidate Key Virulence Genes of V. harveyi at the Early Stage of Infection

During the initial stage of infection, the pathways enriched with significantly up-regulated DEGs in pathogenic bacteria may serve as crucial virulence factors, enabling them to colonize the host [31]. Thus, critical candidate virulence genes were explored through an in-depth analysis of differential gene expression in specific functional pathways.

#### 3.5.1. Siderophore Biosynthesis and Transport

**Figure 4:**
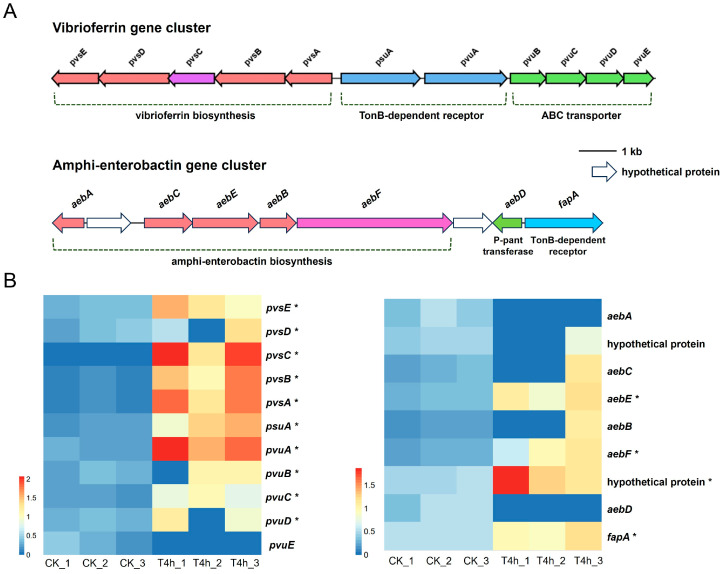
Analysis of siderophore biosynthesis and transporter genes of *V. harveyi* GDH11385. (**A**) The gene organization of two siderophore (vibrioferrin and amphi-enterobactin) gene clusters encoded by strain GDH11385 determined by genome mining. (**B**) Heatmap showing the variations in gene expression of siderophore (vibrioferrin and amphi-enterobactin) biosynthesis and transporter genes of strain GDH11385 in the host compared to expression in pure culture in vitro. The abundance of gene transcripts was represented as log10-transformed TPM values. Red color denoted higher expression levels, while blue indicated lower expression levels. Significantly altered genes were marked with an asterisk (*). Gene names or protein annotations were located on the right side of the heatmap.

The availability of iron within the host is constrained by its binding to proteins such as transferrin and lactoferrin. Thus, bacterial pathogens typically have to deploy siderophores to scavenge iron from the host environment. This contributes to the colonization of pathogens within the host and increases the severity of disease [32]. Genome mining revealed that *V. harveyi* GDH1385 encodes two siderophore gene clusters, which are responsible for the synthesis, uptake, and transport of the carboxylate siderophore vibrioferrin and the triscatecholate siderophore amphi-enterobactin, respectively (Figure 4A). The vibrioferrin gene cluster is constituted of five siderophore biosynthesis genes (*pvsABCDE*), two ton-dependent receptor genes (*psuA* and *pvuA*), and four ATP-binding cassette transporter genes (*pvuBCDE*). It is noteworthy that 10 of the 11 genes in the vibrioferrin gene cluster exhibited a significant increase in expression levels when *V. harveyi* infected the host, as compared to the levels observed in pure culture in vitro (Figure 4B). The second siderophore amphi-enterobactin gene cluster is composed of six genes (*aebABCDEF*) that encode proteins that are homologs to those involved in enterobactin biosynthesis, along with a ton-dependent receptor gene *fapA*. In contrast to the vast majority of genes that were significantly up-regulated in the vibrioferrin gene cluster, only three genes related to amphi-enterobactin synthesis and utilization were significantly up-regulated in *V. harveyi* during the infection of the host (Figure 4B). This suggests that the role of amphi-enterobactin may be less significant than that of vibrioferrin during the early stage of infection.

#### 3.5.2. Type VI Secretion System (T6SS)

**Figure 5:**
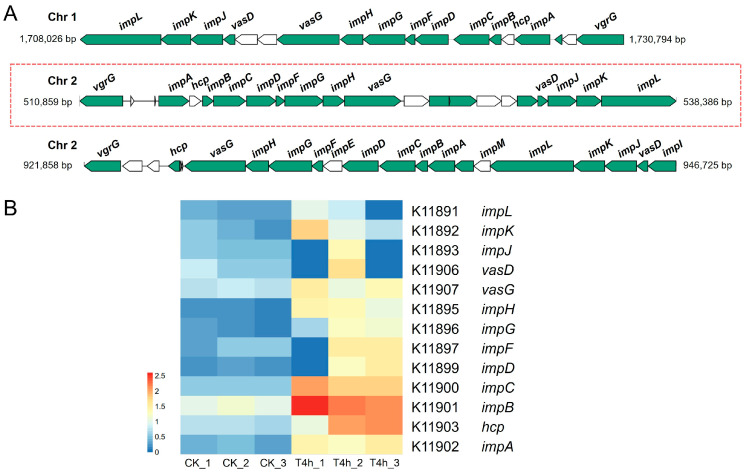
Analysis of type VI secretion system (T6SS)-associated genes of *V. harveyi* GDH11385. (**A**) The gene organization of three T6SS gene clusters encoded by strain GDH11385 determined by genome mining. (**B**) Heatmap presenting the variations in gene expression of one T6SS gene cluster of strain GDH11385 in the host compared to expression in pure culture in vitro. The abundance of gene transcripts was represented as log10-transformed TPM values. Red color denoted higher expression levels, while blue indicated lower expression levels. Gene names or KO IDs were located on the right side of the heatmap.

The bacterial secretion system is a nanomolecular complex that releases a variety of virulence factors into the periphery or translocates them to target host cells. For example, many bacterial pathogens employ the type VI secretion system (T6SS) to facilitate colonization and virulence production in the host [33,34]. As shown in Figure 5A, the genome of *V. harveyi* GDH1385 contains three T6SS gene clusters, with one located on chromosome 1 and two on chromosome 2. The transcriptome data showed that most of the genes in one of the three T6SS gene clusters (chr 2, ranging from 510,859 bp to 538,386 bp) were significantly up-regulated during *V. harveyi* infection of the host. These significantly up-regulated genes include those encoding the ATPase TssH (*vasG*), the baseplate subunit TssG (*impH*), the baseplate subunit TssF (*impG*), the baseplate subunit TssE (*impF*), the contractile sheath large subunit (*impD* and *impC*), the contractile sheath small subunit (*impB*), the tube protein Hcp (*hcp*), and the ImpA family N-terminal domain-containing protein (*impA*) (Figure 5B). The above results suggest that although the pathogen *V. harveyi* GDH1385 possesses multiple T6SS systems, only one of them is associated with virulence towards the host during the early stage of infection.

#### 3.5.3. Flagellar Assembly

**Figure 6:**
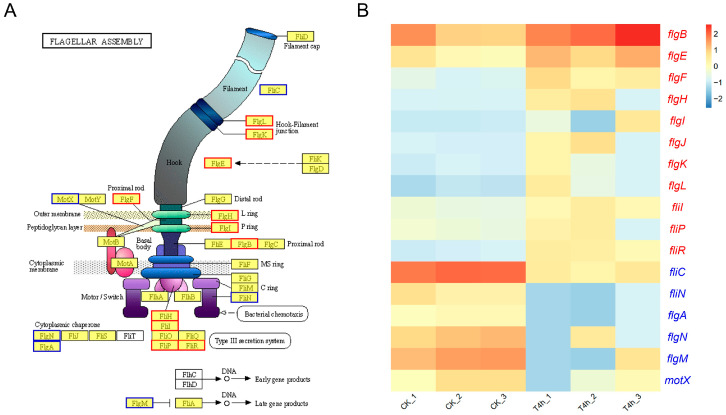
Analysis of genes involved in flagellar assembly in *V. harveyi* GDH11385. (**A**) Schematic representation of the flagellar complex of strain GDH11385 as determined by KEGG pathway mapping. Red borders indicate up-regulated DEGs, and blue borders represent down-regulated DEGs. (**B**) Heatmap illustrating the variations in gene expression of flagellar assembly genes of strain GDH11385 in the host compared to expression in pure culture in vitro. The abundance of gene transcripts was represented as log10-transformed TPM values. Red color denoted higher expression levels, while blue indicated lower expression levels. The red-colored genes on the right side of the heatmap indicate up-regulated DEGs, while the blue-colored genes represent down-regulated DEGs.

The flagellar-mediated motility is closely related to diverse biological processes, such as chemotaxis, biofilm formation, colonization, and virulence in *Vibrio* spp. [35]. The structure of the flagellar complex of *V. harveyi* GDH11385 and the proteins involved in the assembly of these major structures are displayed in Figure 6A. Transcriptome analysis demonstrated that most of the genes involved in flagellar assembly of *V. harveyi* exhibited a significant increase in expression during infection of the host, whereas a small number of genes demonstrated a notable decline in expression during this process. Specifically, the genes associated with flagellar basal body (*flgB*, *flgF*, *flgH*, and *flgI*), rod cap (*flgJ*), hook (*flgE*), hook–filament junction (*flgK* and *flgL*), and type III secretion system (*fliR*, *fliP*, *fliI*, and *fliH*) were found to be significantly up-regulated in strain GDH11385 during host infection. In contrast, the genes encoding flagellin (*fliC*), flagellar motor switch protein FliN (*fliN*), flagellar basal body P-ring formation protein FlgA (*flgA*), flagellar protein FlgN (*flgN*), flagellar biosynthesis anti-sigma factor FlgM (*flgM*), and sodium-type polar flagellar protein MotX (*motX*) exhibited a notable decline in expression in strain GDH11385 within the host (Figure 6B). It can be posited that flagellar-mediated cell motility may function in the virulence of *V. harveyi* at the early stage of infection. However, the contribution of these flagellar assembly-related genes to the pathogenicity of *V. harveyi* and its interactions with hybrid groupers remains to be fully elucidated through further research.

## 4. Discussion

*V. harveyi* has emerged as the predominant *Vibrio* species associated with severe infections and mortality of marine fish in southern China and is therefore considered a major impediment to fisheries production [3,4]. We have recently demonstrated that a novel virulent genotype of *V. harveyi* is circulating in Hainan and Guangdong, which exhibits high virulence to the hybrid grouper [1,8]. Nevertheless, the precise pathogenesis and virulence determinants of this virulent genotype of *V. harveyi* remain poorly understood, largely restrained by their inability to accept exogenous DNA by conjugal transfer, which presents a significant obstacle to related genetic manipulations [36]. The application of dual RNA-seq methodology for the surveillance of transcriptional changes of bacterial pathogens within the host tissue presents a novel strategy for the identification of critical virulence genes involved in the process of bacterial invasion [18,20,37].

In this work, we developed an infection model of the hybrid grouper infected with *V. harveyi* and performed RNA-seq analysis on liver samples of the hybrid grouper injected with *V. harveyi* at 4 hpi. This was performed to investigate the transcriptional changes of *V. harveyi* in vivo within the host environment as compared to that of pure culture in vitro. The transcriptome data revealed that more than one-quarter of the genomic genes of *V. harveyi* exhibited significantly different expression patterns in the host at 4 hpi in comparison to pure culture in vitro, suggesting that *V. harveyi* is highly responsive to the host at the initial stage of infection. Furthermore, the number of genes with down-regulated expression exceeded the number of genes with up-regulated expression (Figure 2), a finding consistent with that observed in multiple models of bacteria–host infection interactions [20,30,38]. It is hypothesized that this was due to the extremely oligotrophic host environment, which compelled bacterial pathogens to adjust their metabolic and physiological processes. This was evidenced by the fact that the majority of the down-regulated DEGs were enriched in pathways or processes related to metabolism (Figure 3).

Bacterial pathogens require a number of coordinated virulence factors to evade the host’s immune system, achieve colonization, and subsequently proliferate within the host [39]. KEGG enrichment analysis demonstrated that *V. harveyi* was significantly enriched for several virulence-related pathways at the early stage of infection, with the siderophore synthesis and transport pathway being the most highly enriched, and in particular almost all of the genes associated with vibrioferrin synthesis and transport were significantly up-regulated (Figure 3A and Figure 4). Iron metabolism is of pivotal importance during bacterial infections. It has previously been reported that infection with *V. harveyi* strain 345 attacked the liver, a central immune organ in the Pearl Gentian Grouper, probably causing iron limitation and thus pathological damage to the host [10]. Siderophores are low-molecular-weight metal chelators that facilitate the high-affinity transport of iron, thereby overcoming environmental and host iron limitations for survival [40]. In addition to iron acquisition, recent studies have revealed the noncanonical roles of siderophores, e.g., contributing to host pathogenicity by interacting with host cells to produce proinflammatory effects, participating in biofilm formation, and aiding in resistance to oxidative stress through the removal of ROS [41,42]. It can thus be proposed that the significant up-regulation of the siderophore vibrioferrin synthesis and transport pathway by *V. harveyi* during the initial stage of infection represents a strategy to facilitate iron acquisition and utilization, thereby enhancing host colonization.

The T6SS system has been demonstrated to mediate the interaction with eukaryotic cells by delivering various anti-eukaryotic effectors that can affect membrane integrity, manipulate the host cytoskeleton, or perturb host innate immunity and other host responses [43,44]. The bacterial secretion system pathway was found to be significantly enriched in *V. harveyi*, with most of the genes within one of the three T6SS gene clusters exhibiting a significant increase in expression (Figure 5B). It is not uncommon for a single bacterial genome to contain multiple T6SS gene clusters; e.g., *Pseudomonas aeruginosa* encodes three distinct sets of T6SS, *V. parahaemolyticus* possesses two T6SS gene clusters, and *Burkholderia pseudomallei* harbors up to six T6SS gene clusters. It is worth noting, however, that the functions of these T6SS gene clusters are not usually redundant in a strain [45,46,47]. To the best of our knowledge, the specific role of T6SS in the pathogenesis of *V. harveyi* has yet to be documented. Hence, the identification of the up-regulated T6SS gene cluster in the present study offers a more specific target for elucidating the virulence mechanism of *V. harveyi* at the early stage of grouper infection.

Flagellar-mediated motility contributes to the virulence of pathogenic bacteria through chemotaxis and adhesion to and invasion of host surfaces [48,49]. A transcriptomic study has recently shown that the flagellar assembly pathway is crucial for the adaptation of *V. harveyi* strain TS-628 from seawater to the muscle of the host banded grouper (*Epinephelus awoara*) [50]. The bacterial flagellum typically consists of several major elements, including the passive (non-motor) structural elements (basal body, hook, filament), the motor (rotor/switch element or C ring, stator, or Mot complexes), and the type III flagellar protein export apparatus [51]. The results of this study showed that the expression of most of the genes associated with the structural elements of the flagellum, including the basal body, rod cap, hook, and hook–filament junction, was significantly increased in strain GDH11385 during the early stage of host infection. Furthermore, genes involved in the type III secretion system, which is responsible for flagellar protein export, also demonstrated a significant increase in expression. The above results suggest that these genes may be critical for the initial adhesion of *V. harveyi* to hybrid grouper.

In summary, the transcriptome expression profiles of *V. harveyi* in the host tissue were analyzed using dual RNA-seq, which revealed distinct gene expression patterns of *V. harveyi* in vivo and in vitro of the hybrid grouper. The findings of this study have led to the identification of potential key virulence genes at the early stage of infection, thereby paving the way for more focused investigations into the molecular virulence mechanisms of *V. harveyi*. Future research should focus on the transcriptional changes in *V. harveyi* at additional time points post-infection, as well as the transcriptional responses of the host, which are crucial for gaining a deeper insight into bacteria–host interactions. This is of great significance for the development of effective prevention and control strategies against this pathogen.

## Figures and Tables

**Table 1:** Summary of transcriptome data.

Sample	Clean Reads	Clean Q20 (%)	Clean Q30 (%)	Genome Mapped Reads (Ratio)	Uniq Mapped Reads (Ratio)
T4h_1	80,594,036	97.42	94.12	50,456 (0.06%)	29,468 (0.04%)
T4h_2	73,525,504	97.46	94.09	53,577 (0.07%)	28,826 (0.04%)
T4h_3	82,791,536	97.51	94.15	56,965 (0.07%)	29,169 (0.04%)
CK_1	27,122,138	98.52	95.48	26,718,117 (98.51%)	24,604,823 (90.72%)
CK_2	27,680,952	98.52	95.51	27,279,620 (98.55%)	25,026,703 (90.41%)
CK_3	27,241,302	98.46	95.36	26,855,857 (98.59%)	24,916,543 (91.47%)

T4h represents the 4 hpi liver sample collected from infected groupers, while CK corresponds to the in vitro cultured bacterial control. Each experimental group consisted of three replicates. The sequencing results were mapped to the reference genome of *V. harveyi* GDH11385.

## Data Availability

The original contributions presented in the study are included in the article, further inquiries can be directed to the corresponding author.
